# First Report on Mycotoxin Contamination of Hops (*Humulus lupulus* L.)

**DOI:** 10.3390/toxins16070293

**Published:** 2024-06-26

**Authors:** Ivana Dodlek Šarkanj, Nada Vahčić, Ksenija Markov, Josip Haramija, Natalija Uršulin-Trstenjak, Krunoslav Hajdek, Michael Sulyok, Rudolf Krska, Bojan Šarkanj

**Affiliations:** 1 Department of Food Technology, University North, Trg dr. Žarka Dolinara 1, HR-48000 Koprivnica, Croatia; idsarkanj@unin.hr (I.D.Š.); natalija.ursulin-trstenjak@unin.hr (N.U.-T.); 2 Faculty of Food Technology and Biotechnology, University of Zagreb, Pierottijeva 6, HR-10000 Zagreb, Croatia; nvahcic@pbf.hr (N.V.); ksenija.markov@pbf.hr (K.M.); 3 Koprivnica Branch, State Inspectorate, Florijanski trg 18, HR-48000, Koprivnica, Croatia; josip.haramija@dirh.hr; 4 Department of Packaging, Recycling and Environmental Protection, University North, Trg dr. Žarka Dolinara 1, HR-48000 Koprivnica, Croatia; khajdek@unin.hr; 5 Institute of Bioanalytics and Agro-Metabolomics, Department of Agrobiotechnology (IFA-Tulln), University of Natural Resources and Life Sciences, Vienna, Konrad Lorenz Str. 20, AT-3430 Tulln, Austria; michael.sulyok@boku.ac.at (M.S.); rudolf.krska@boku.ac.at (R.K.); 6 Institute for Global Food Security, School of Biological Sciences, Queen’s University Belfast, University Road, Belfast BT7 1NN, UK

**Keywords:** hops, *Humulus lupulus* L., mycotoxins, *Alternaria* spp., *Fusarium* spp., LC-MS/MS

## Abstract

The presence of mycotoxins and other toxic metabolites in hops (*Humulus lupulus* L.) was assessed for the first time. In total, 62 hop samples were sampled in craft breweries, and analyzed by a multi-toxin LS-MS/MS method. The study collected samples from craft breweries in all of the Croatian counties and statistically compared the results. Based on previous reports on *Alternaria* spp. and *Fusarium* spp. contamination of hops, the study confirmed the contamination of hops with these toxins. *Alternaria* toxins, particularly tenuazonic acid, were found in all tested samples, while *Fusarium* toxins, including deoxynivalenol, were present in 98% of samples. However, no *Aspergillus* or *Penicillium* metabolites were detected, indicating proper storage conditions. In addition to the *Alternaria* and *Fusarium* toxins, abscisic acid, a drought stress indicator in hops, was also detected, as well as several unspecific metabolites. The findings suggest the need for monitoring, risk assessment, and potential regulation of *Alternaria* and *Fusarium* toxins in hops to ensure the safety of hop usage in the brewing and pharmaceutical industries. Also, four local wild varieties were tested, with similar results to the commercial varieties for toxin contamination, but the statistically significant regional differences in toxin occurrence highlight the importance and need for targeted monitoring.

## 1. Introduction

Hops (*Humulus lupulus* L.) are an essential ingredient in brewing, imparting a bitter flavor and floral aroma to beer. The first written record of the utilization of hops in brewing dates back to 736 in a monastery document from the Hallertau region in Bavaria, Germany [1]. Since then, hops have been well documented for their role in flavoring, preserving, and stabilizing beer. The female inflorescences of the hop plant, known as cones or strobili, are particularly valued for their production of secondary metabolites, such as terpenes, sesquiterpenes, and prenylated phenolic compounds, which contribute to the beer’s bitterness and aromatic qualities [2]. These compounds also exhibit antiseptic properties, enhancing the beer’s shelf life and safety [3]. However, the cultivation and storage of hops is not without microbial challenges. One significant concern is the contamination of hops with (mycotoxigenic) fungi [4], which can produce mycotoxins that pose health risks to consumers. Mycotoxins are toxic secondary metabolites produced by certain species of fungi [5], and their presence in food and beverages is a serious safety issue, particularly in a changing climate [6]. The biological control of these fungi and their toxins is a critical area of research, with studies focusing on pre-harvest approaches to mitigate the risk of contamination [7], and also ensuring stability and prevention of infection during storage [8]. Hops have been shown to be contaminated by all the main genera of mycotoxin-producing fungi [4]: *Alternaria* spp., *Aspergillus* spp., *Fusarium* spp., and *Penicillium* spp. [9]. There are several reports of *Alternaria alternata* infections of hops [4,10,11,12], a known producer of *Alternaria* toxins, recently regulated in the EU (EC 553/2022 [13]). The infection symptoms include necrotic lesions on the tips of bracts and bracteoles of developing cones [14]. The disease is called *Alternaria* cone disorder (ACD) and is widespread in hop yards and other agricultural systems worldwide. There is still no reported occurrence of any of the *Alternaria* toxins in hops, although their presence has been implied due to frequent infection reports. *Aspergillus* spp. is one of the fungi genera that has still not been isolated in naturally infected hops [4]; moreover, hop extracts have shown great antifungal properties against *Aspergillus* spp. [15,16]. *Fusarium* spp. is one of the most often reported mycotoxin-producing species in hops. It is known to cause hop fusariosis (HF) [17], *Fusarium* canker (FC) wilting, cankers in the crown, foliar necrosis and death of infected plants [18], and hop wilt (HW) [19]. Several *Fusarium* species have been identified as the causal agents of HW, including *F. oxysporum*, *F. culmorum*, *F. solani*, *F. proliferatum*, and *F. acuminatum* [19]. *F. oxysporum* and *F. culmorum* were most frequently isolated, and both are known mycotoxin producers [20,21]. The *Fusarium* fungi first colonize the underground plant parts (roots, crown, and rootstocks) and the basal part of the stem, from where they disperse and attack the neighboring vascular tissues. The interrupted delivery of water and nutrients to the terminal plant parts causes chlorosis, necrosis, and wilting, first of the apical leaves, and then, of the lower leaves. Due to the relatively high occurrence of *Fusarium* infection, there have also been qualitative and quantitative PCR assays developed for the detection of *Fusarium* spp. in hops [22]. Although a high incidence of *Fusarium* fungi is well documented, no data on the occurrence of *Fusarium* mycotoxins have been published. The growth of *Penicillium* spp. has rarely been reported on hops. It has been shown that hop extracts inhibit the growth of several *Penicillium* spp. [15,16,23] but, on the other hand, *Penicillium* spp. was isolated as a probable source of diastatic enzymes driving “hop creep” in dry-hopped beer [24,25]. After identification of the presence of *Penicillium* spp., there were no data on the presence of their mycotoxins in hops. Interestingly, hop essential oils and extracts possess antifungal properties, which can be leveraged to combat the growth of mycotoxigenic fungi [26]. Isoxanthohumol, an isoprene flavonoid found in hops, has demonstrated significant antifungal activity against phytopathogenic fungi such as *Botrytis cinerea*. This compound disrupts the metabolic processes of the fungi, affecting their carbohydrate metabolism and hindering ATP generation by inhibiting respiration. Additionally, isoxanthohumol causes membrane lipid peroxidation, accelerating the death of fungal cells. These findings suggest that hops not only contribute to the sensory qualities of beer but also have the potential to enhance its safety by reducing the risk of fungal contamination [27]. In addition, extracts of *H. lupulus* inhibited the mycelial growth of *F. culmorum*, a pathogenic fungus that causes root rot in wheat [28]. The oils and extracts of *H. lupulus* also showed activity against the fungus *Trichophyton mentagrophytes* var. *interdigitale* [27]. There is a lack of testing for mycotoxin occurrence, although there is enough evidence that mycotoxin-producing fungi can contaminate the hops. The purpose of this study was to investigate the possibility of contamination of the hops by mycotoxins, due to sufficient evidence of the presence of mycotoxin-producing fungi in the published literature. The results constitute additional research in the field and can contribute to the development of risk assessments for hop products, and possible changes in hop production, processing, and storage to cope with a mycotoxin contamination threat. Also, by using a wide range of state-of-the-art multi-toxin methods, additional information on the presence of plant, bacterial, and unspecific toxins are tested, for further risk assessment and increased food safety.

## 2. Results and Discussion

**Figure 1:**
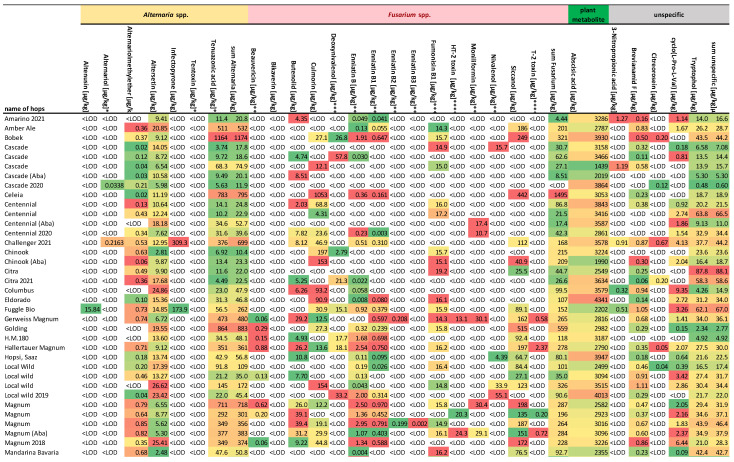

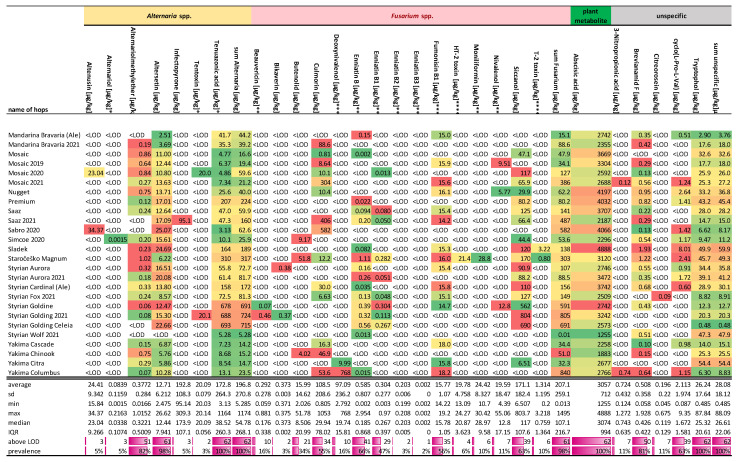
Results of all detected toxins and metabolites in the hop samples. * Toxins mentioned in EC 553/2022 [13]; ** toxins from EFSA’s annual call for continuous collection of chemical contaminants occurrence data in food and feed; *** toxins mentioned in EC 915/2023 [29]; **** toxins mentioned in EC 165/2013 [30].

**Figure 2:**
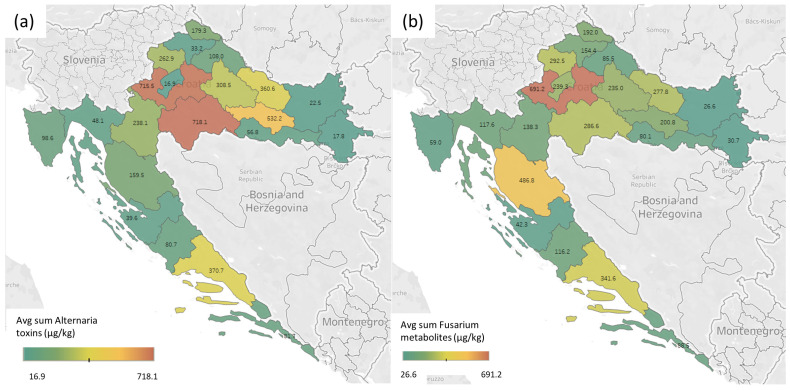

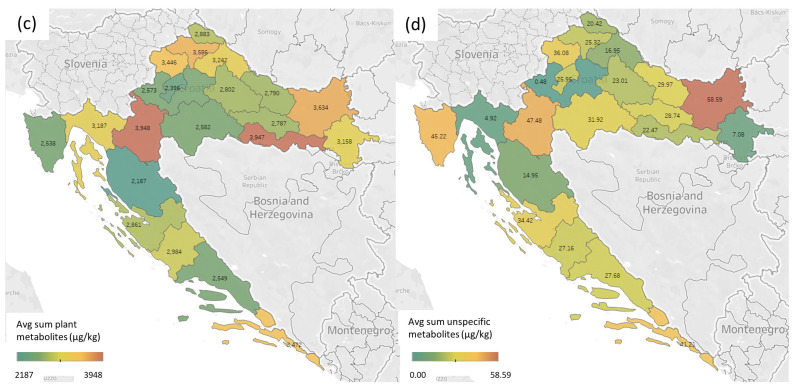
Heat map of the toxin distribution in hop samples from different Croatian counties. All concentrations are the average sum of the toxins in the selected county expressed in µg/kg. The division of the figure is (**a**) *Alternaria* toxins, (**b**) *Fusarium* toxins, (**c**) plant metabolites, (**d**) unspecific metabolites.

The results of this study confirmed the presence of multiple mycotoxins associated with the fungal genera previously detected in hop samples [4,10,11,12,14,17,18,19,21]. The results are divided by the main producers of the toxins/metabolites. A summary of all the results is given in Figure 1 and they are also divided by the producers. All of the collected samples were used in craft breweries in Croatia and four local wild varieties were collected in the market. The data were also used for creating a heat map (Figure 2) and comparing the data between counties by using Kruskal–Wallis ANOVA. All the results showed that there was a statistically significant (*p* < 0.05) difference between the distributions of the data in different counties.

### 2.1. Alternaria Toxins

*Alternaria alternata*, a known producer of *Alternaria* toxins, is one of the fungi that contaminates hop plants [4,10,11,14]. The results in Figure 1 and Figure 2a confirm the contamination of hops with *Alternaria* toxins. All tested samples confirmed the presence of at least one *Alternaria* toxin, with tenuazonic acid (TeA) showing the highest occurrence (100% of the samples), followed by altersetin (ALT) (98% of the samples), and alternariol methyl ether (AME) (quantified in 82% of the samples). The rest of the *Alternaria* toxins were present in less than 5% of the samples—infectopyrone, altenusin, tentoxin (TTX), and alternariol (AOH). A similar occurrence pattern of *Alternaria* toxins was found in similar matrices such as plants from India [31], tobacco [32], and pu-erh tea [33], while different occurrences were found in matrices such as figs, sunflower seeds, wine [34], barley [35], and millet [36].

The occurrence of TTX was low compared to other matrices, both in terms of occurrence data and measured concentrations, when compared to AOH, AME, or TeA. While some studies show higher AOH levels than AME in wheat-based products [37], TeA is always higher in concentration and occurrence compared to other *Alternaria* toxins. Overall, their occurrence data are well summarized in EFSA’s dietary exposure assessment to *Alternaria* toxins in the European population [38]. It is well documented that *Alternaria* spp. produces host-specific mycotoxins [39,40,41], so every matrix will have its specific distribution of different *Alternaria* toxins. Since this is the first report, it is not possible to compare the results to other published results.

In the European Union, three *Alternaria* mycotoxins are recommended for monitoring (EC 553/2022) [13] but not in matrices such as hops, probably due to a lack of data on their occurrence. With these new data, they could be included in a monitoring plan, risk assessment by EFSA, or legislation due to a relatively high maximum concentration of TeA (1174 µg/kg), exceeding most of the proposed limits in the Commission recommendations (EC 553/2022) [13] on monitoring the presence of *Alternaria* toxins in food. The concentrations of the other two *Alternaria* toxins mentioned in the legislation do not exceed the proposed limits, although they should not be excluded from monitoring due to their toxicities [42]. When comparing the locations of the sampling, the highest average sums of all *Alternaria* toxins are in Zagreb County and Sisak-Moslavina County, while the lowest average sums of all *Alternaria* toxins were measured in samples collected in Zagreb City and Vukovar-Srijem County (Figure 2a). When comparing the results, there was a statistically significant difference in the individual and sum of all *Alternaria* toxin distributions between different Croatian counties (*p* < 0.01).

### 2.2. Fusarium Toxins

*Fusarium* spp. is one of the fungi occurring most often on hop plants, causing HF [16], FC (wilting, cankers in the crown, foliar necrosis, and death of infected plants) [17], and HW [18]. The frequent reports of *Fusarium* contamination and no mycotoxin occurrence data were a bit surprising. In this research, we confirmed the presence of 15 different *Fusarium* toxins, and on average 98% of the samples were contaminated by at least one of the *Fusarium* mycotoxins (Figure 1 and Figure 2b). Out of the quantified mycotoxins, some are regulated in the European Union (deoxynivalenol (DON), fumonisin B1 (FB1) by commission regulation EC 915/2023 [29]); followed by some regulated by Commission recommendations on the presence of T-2 and HT-2 toxins in cereals and cereal products (EC 165/2013 [30]) (T-2 and HT-2 toxins); mycotoxins from EFSA’s annual call for continuous collection of chemical contaminants occurrence data in food and feed (beauvericin (BEA), enniatin B (ENNB), enniatin B1 (ENNB1), enniatin B2 (ENNB2), enniatin B3 (ENNB3), moniliformin (MON), nivalenol (NIV)), and others are not mentioned in any official document. Interestingly, no modified or masked forms of mycotoxins were found, possibly due to relatively low contamination of specific metabolic pathways in hops, which do not include glucuronidation and sulfation. UDP-glucosyltransferase (UGT) is used in plants for modifying/masking of mycotoxins [9], but in hops it can also interfere with the taste through the formation of flavorless glucosides of terpenoids [43]. Therefore, hop varieties used in brewing are usually ones with lower UDP activity, diminishing their capacity for defending against mycotoxins. Although we also tested four local wild varieties, they also did not show any ability of masking compared to the commercial varieties.

Out of the unregulated *Fusarium* mycotoxins, the presence of bikaverin (BKV), butenolide (BUT), culmorin (CUL), and siccanol (SIC) was confirmed. When comparing the occurrence of *Fusarium* mycotoxins, ENNB had the highest occurrence, detected in 66% of samples, followed by SIC (63%), FB1 (56%), CUL (55%), ENNB1 (47%), BUT (34%), BEA (16%), DON (16%), NIV (11%), MON (10%), T-2 (10%,), HT-2 (6%), BKV (3%), ENNB2 (3%), and ENN B3 (2%). When comparing the regulated mycotoxins to their legal limits, the highest detected DON level (768 µg/kg) exceeds limits for baby food and processed cereal-based food for infants and young children, bread, pastries, biscuits, cereal snacks and breakfast cereals, cereals placed on the market for the final consumer, cereal flour, semolina, bran and germ as final products placed on the market for the final consumer, and other milling products of maize not placed on the market for the final consumer. With this detected level it is advisable to perform widespread screening and additional risk assessment, due to hops’ usage in mainly water-soluble products (beer, infused water, and pharmaceutical products). Comparing the T-2 and HT-2 levels to the legislation, all samples with detected HT-2 toxin (with a maximum of 3.22 µg/kg and 24.3 µg/kg, respectively, and a median of 0.76 µg/kg and 15.8 µg/kg) exceeded the indicative level for cereal-based foods for infants and young children, and one sample exceeded the level for bread (including small bakery wares), pastries, biscuits, cereal snacks, and pasta. The FB1 levels were quite low compared to the legal limits, with a maximum detected level of 19.2 µg/kg, and median level of 15.8 µg/kg. Out of the other mycotoxins, the presence of enniatins (ENNs) was expected, and they were present in low concentrations, although their co-occurrence was high (all ENNs and BEA). The high occurrence of CUL (55%), and its high detected concentration of up to 1053 µg/kg, a *Fusarium* mycotoxin that suppress the in vitro glucuronidation of DON, can be concerning, due to lowering humans’ detoxification capacity towards DON [44]. The other two unregulated, yet highly occurring, mycotoxins were SIC and BUT, with 63% and 55% occurrence in the tested samples. Their highest detected concentrations were 804 µg/kg and 51.8 µg/kg, but since there are not enough toxicological data at these concentrations, their toxicological relevance is yet to be investigated. Similar co-occurrence was not detected in other matrices, leaving the possibility of a similar host-dependent pattern of mycotoxins as found in *Alternaria* toxins [39,41], and also for *Fusarium* mycotoxins [45,46]. When comparing the regional distribution of the *Fusarium* toxins in hops, the same counties (and samples) that were highly contaminated by *Alternaria* toxins were also highly contaminated by *Fusarium* toxins. This is possible due to the phytotoxic effect of TeA, as it helps *Alternaria* spp. spread on the plants [47]. When compared, there was a statistically significant (*p* < 0.05) correlation between TeA and the sum of *Alternaria* toxins (r = 0.98), and TeA and the sum of *Fusarium* toxins (r = 0.61). Zagreb county had the highest average contamination by the sum of *Fusarium* toxins (691 µg/kg), while Osijek-Baranja and Vukovar-Srijem counties had the lowest average measured concentrations of *Fusarium* toxins (Figure 2b), similarly to *Alternaria* toxins (Figure 2a).

### 2.3. Plant Metabolites

The only plant metabolite that was detected in quantifiable concentrations was abscisic acid (ABA), as shown in Figure 1. It was detected in all of the samples at relatively high concentrations, ranging from 1255 µg/kg to 4888 µg/kg. As one of the drought stress indicators, ABA was surprisingly not correlated with a lot of the mycotoxins, although mycotoxin production is correlated with drought during growth [6,48,49]. The only statistically relevant correlations (*p* < 0.05) were between ABA and NIV (r = 0.26), and ABA and ALT (r = 0.41). Also, in regional distribution (Figure 2(c)), the average ABA concentrations were not similar to other mycotoxins, with the highest values in Karlovac and Slavonski Brod Posavina County (3948 µg/kg and 3947 µg/kg), and the lowest in Lika-Senj County and City of Zagreb (2187 µg/kg and 2396 µg/kg). The detected ABA concentrations are affected by the growth conditions and are not expected to change significantly during storage.

### 2.4. Unspecific Metabolites

The unspecific metabolites are all the other metabolites that can be produced by several fungal, bacterial, or plant sources. The most common was tryptophol, often found in yeast as a quorum-sensing molecule [50], which can be produced by plants, bacteria, fungi, and sponges [51] (Figure 1). In the tested hop samples, it was the most frequently occurring unspecific metabolite, with a range from 0.48 µg/kg to 88.1 µg/kg, and a median of 25.3 µg/kg. The second most frequently occurring metabolite was brevianamide F or cyclo-(L-Trp-L-Pro), found in different microorganisms as a precursor of tryptophan-proline 2,5-diketopiperazines, a large group of primary and secondary metabolites in microbes, that explains the high occurrence of 81%, with relatively low concentrations, from 0.06 µg/kg to 1.93 µg/kg. Also, in addition to this cyclopeptide, cyclo-(L-Pro-L-Val) was also often detected (in 63% of the samples), but also at similar low concentrations, from 0.09 µg/kg to 9.35 µg/kg. The last two of the unspecific metabolites were 3-nitro propionic acid and citreorosein, both in 11% of the samples, with relatively low ranges (0.12–1.27 µg/kg and 0.04–0.67 µg/kg, respectively). Both of these metabolites can be produced by a wide variety of fungi, and at the detected concentrations they do not pose a risk to human health. Interestingly, when comparing the regional distribution, the highest average concentration of the sum of unspecific metabolites was in Osijek-Baranja County, while the lowest was in Zagreb County, the reverse of the other fungal metabolites (*Alternaria* and *Fusarium*) (Figure 2d).

It is interesting to note that no *Aspergillus* or *Penicillium* metabolites were detected. Both fungal genera are considered storage fungi, suggesting that all craft brewers that were storing the hops for brewing were careful about the storage conditions. It is recommended by the producers that hops are stored in a cold, dark, and dry environment, such as a freezer, where all chemical reactions that degrade hops’ quality are slower. On the other hand, it was expected, according to the literature data, that there should not be any *Aspergillus* spp. and a small amount of *Penicillium* spp., and this was confirmed by the metabolite scan.

### 2.5. Transfer of Mycotoxins in Hop Products

The major usage of hops is in the brewing industry, where they can be used in three forms: whole dried cone, pellets, and hop extract. The largest worldwide producer is the European Union, with an annual production of 50,000 tons, where the main producers are Germany, Czechia, Poland, and Slovenia [52]; and other high producing countries are the United States, China, and Australia, according to Statista data for the production volume of hops worldwide in 2022 by country [53]. Currently, more than 98% of all produced hops are used in the brewing industry, where the main product used for brewing is hop pellets [1], which have been investigated in this research. Outside the brewing industry, the rest of the usage is in the pharmaceutical industry, where they can use fresh hops or spent hops—a by-product of the brewing industry. As summarized by Korpelainen, et al. (2021), traditionally, hops were used in beer flavoring, for preserving and clarifying, as a vegetable, in bread making (to cultivate yeast), as a preservative in sausages, for flavoring water, in baked goods, in tobacco, as cattle fodder, for manure preparation, as a hair rinse for brunettes, as a deodorant (antimicrobial, fragrance), in perfumes, in skin lotions, as oil, in pharmacy as an antibiotic, as an anti–inflammatory, as a sedative, for sleep disturbances, headache, restlessness, tenderness of limbs, bleary eyes, gastric problems, indigestion, appetite, toothache, earache, neuralgia, for treating leprosy, tuberculosis, asbestosis, and silicosis, as an anthelmintic, as an antiparasitic drug, for cough, spasms, fever, and anxiety, for clearing blood, flatulence, delirium tremens, irritable bladder, aches, and diuresis, and in liver disorders (porphyria) [1].

The effects of processing on mycotoxins are diverse based on the type of processing. Generally, mycotoxins are considered thermostable, and that is why they are one of the most critical of the chemical contaminants in processed food [5]. Ninety-eight percent of produced hops are used in the brewing industry. The first step in hop processing is drying, which has to be performed gently at low temperatures (below 60 °C) to prevent color and flavor changes [1]. At these temperatures mycotoxins are stable, and no mycotoxin losses are expected [5]. After drying, the next step is milling and palletization, where low temperature extruders are used. The palletization temperature should not exceed 50 °C to reduce color and flavor changes [1]. There are no data on mycotoxin loss in the extrusion process at such low temperatures, but Janić Hajnal et al. (2022) have investigated the effects of the extrusion process on *Fusarium* and *Alternaria* mycotoxins (the two most relevant mycotoxin groups found in this research) in triticale flour [54] and concluded that due to complex interaction of various parameters, the effect of the extrusion process on the investigated mycotoxins still needs to be determined in detail for each combination of ingredients as well as for the applied parameters. Use of the optimal parameters determined for lowering the concentrations of the investigated mycotoxins gave a 9.5–85.7% reduction. Based on those results, it can be expected that raw hops have a higher mycotoxin content prior to palletization, but this presumption should be confirmed in a separate study. Finally, mycotoxin transfer from hops to beer would be expected to be highly dependent on the type of hopping during brewing and the chemical characteristics of the transferred mycotoxin. It is expected that all mycotoxins that are polar (e.g., DON) should be transferred to the final product, with a reduction by binding to yeast cells during fermentation, or masking due to the metabolic activity of yeast [9]. Hops can be added early during boiling, and all the way towards the end of fermentation. The contact time and temperature would change the mycotoxin transfer from the hops to the beer. This transfer has been noted in some research, where the authors had new, previously undetected mycotoxins in beer that were not found in cereals used in brewing or in wort, but were detected after hopping [55]. It is highly possible that those mycotoxins were extracted from hops. Mastanjević et al., (2019) detected statistically significant higher DON concentrations in beer samples than in wort [56], while in other research a decrease in the mycotoxin content between the wort and the beer was noted, except for zearalenone and tentoxin, the concentrations of which increased without explanation [57]. None of the published research prior to this paper considered hops as the source of the mycotoxins. Still brewing is a complex process that can have unexpected and unpredicted changes in the mycotoxin mixture from the basic ingredients to the final beer, depending on numerous variables—contamination of the ingredients, parameters used during brewing, yeast used during fermentation, used equipment, and type of beer [55]. It is not possible to find mycotoxin-free samples of cereals and hops to be able to eliminate one variable in mycotoxins transfer to the final product, and the only possible reliable way the transfer could be calculated would be by adding a known amount of mycotoxins by spiking samples and comparing them to unspiked ingredients under the same brewing conditions, or even more precisely, the usage of stable-isotope-labeled mycotoxin standards, that are currently not available for all of the toxins that have been detected in cereals and hops.

### 2.6. Health Perspective, Limitations, and Outlook

Out of the detected mycotoxins, the most concerning for health are those that are mentioned in the legislation such as AOH, AME, TeA, DON, FB1, T-2, and HT-2, and those that EFSA recognized in the call for continuous collection of chemical contaminant occurrence data in food and feed such as TTX, ENNs MON, and NIV. There are many reports on the toxic effects of the mentioned mycotoxins. AOH, AME, TeA, and TTX were found in relatively low concentrations compared to those that were investigated in toxicity testing studies [38,58]. Aichinger et al. (2021) have reviewed the toxic effects of *Alternaria* toxins with the available data, and AOH and AME, based on EFSA’s toxicological threshold of concern (TTC) values, showed increased risk compared to TeA. TeA has shown moderate acute toxicity in in vivo and in vitro tests, while AOH showed immunosuppression and topoisomerase poisoning in in vitro tests, and estrogenicity and androgenicity (endocrine disruption) in in silico and in vitro tests [58]. The toxic effects of *Fusarium* toxins are well documented, where trichothecenes have been studied in more detail, due to their higher occurrence compared to other *Fusarium* mycotoxins [5,9]. There are proposed values of TDI for DON (1 μg/kg b.w./day) based on the reduced body weight gain of mice, and an acute reference dose (ARfD) of 8 μg/kg b.w. per meal was calculated [59]; the value for FB1 was 1 μg/kg b.w./day based on the increased incidence of megalocytic hepatocytes in the chronic study in mice [60]; as well as a combined temporary TDI for the sum of T-2 and HT-2 of 0.06 μg/kg b.w./day based on the general toxicity, hematotoxicity, and immunotoxicity of T-2 toxins [61]. Since, currently, hops are mainly used for beer flavoring, the detected concentrations should not pose a risk to human health if ingested in beer, and there are other ingredients that pose a greater threat (malt, cereals) to the mycotoxin burden of the final beer. The potential hazard could be in the application of the hops as a pharmaceutically active substance, where they are applied differently and can be absorbed by the body through the skin or intravenously. Additionally, risks should be calculated for the farmers and hop processing plant workers that could inhale the hop dust containing microbes and mycotoxins. The additional concerns are due to co-occurring mycotoxins that can change the total toxic impact; one of these highly occurring combinations that was noted in hops is DON-CUL. Culmorin can suppress the in vitro glucuronidation of DON, a main route of detoxification of DON in animals [44]. In combination, CUL can decrease animals’ potential to detoxify DON and increase its toxicity. There are still numerous uninvestigated combinations that could have antagonistic, additive, or synergistic effects.

The limitations of this study are the single year of monitoring, where only one season of weather that could affect the mycotoxin occurrence is captured; for further perspectives, it would be good to continue with regular monitoring to confirm the occurrence pattern. Additionally, it would be interesting to check if the same mycotoxin pattern is observed worldwide, or only in European hop samples. This first-report study, while contributing new insights, also underscores the need for further research to corroborate these initial results. In further research, the extraction patterns should also be evaluated due to differences in hop usage in the brewing industry (there are different times and temperatures of hop addition, that can affect the extraction of mycotoxins), or when used in the pharmaceutical industry different solvents can be used (water, vaseline). In brewing, bittering hops can be added early in the boil, flavor hops are added in the middle towards the end of the boil, aroma hops are added at the very end of the boil, or just after the boil, dry-hopping is performed during or after fermentation, usually cold, and whirlpool hopping is performed during the whirlpooling process. With an increased number of reports, a proper risk assessment can be calculated and it can be assessed whether changes in legislation are needed to ensure human health.

## 3. Conclusions

This study is the first one to confirm the presence of multiple mycotoxins associated with fungi in hop samples, indicating the need for additional risk assessment. Notably, Alternaria toxins were prevalent, with tenuazonic acid showing the highest occurrence (in all tested samples). Fusarium toxins were also widespread, posing concerns due to their regulatory limits, especially DON, which exceeded limits for various food products. The correlation between the distribution of Alternaria and Fusarium toxins suggests a shared contamination pattern in the field. These results highlight the importance of monitoring and regulating mycotoxin levels in hops to ensure consumer safety in craft brewery products.

## 4. Materials and Methods

A total of 62 hop samples used by Croatian craft breweries were collected, with at least one sample per county, so the whole Croatian market was included. The samples were collected in winter 2022 and spring 2023 in accordance with the Commission Regulation (EC) No 401/2006 of 23 February 2006, laying down the methods of sampling and analysis for the official control of the levels of mycotoxins in foodstuffs, with the help of official inspectors and trained scientists for the sampling. All of the hop samples collected were from the harvesting season of 2021 and 2022, and were in the form of pellets. Brewers (where samples were collected) kept the hop samples tightly closed or in the original packaging with zip locks and stored them in freezers (−18 °C) or refrigerators (+4 to +8 °C) to keep the aroma profile of the hops. The samples were stored and transported to Austria at −20 °C. For the extraction and quantitation, the LC-MS/MS multi-mycotoxin method developed by Sulyok et al. (2020) [62] was used.

### 4.1. Chemicals and Reagents

Acetonitrile, methanol, and glacial acetic acid (MS grade) were purchased from Merck (Darmstadt, Germany), while ammonium acetate was purchased from Sigma-Aldrich (Vienna, Austria). Ultra-pure water was produced by Elga Purelab ultra from Veolia Water (Bucks, UK). The standards used for calibration of the multi-toxin LC-MS/MS method were obtained either as gifts from various research groups, or from commercial sources including AnalytiCon Discovery (Potsdam, Germany), Axxora Europe (Lausanne, Switzerland), BioAustralis (Smithfield, Australia), Enzo Life Sciences (Lausen, Switzerland), Iris Biotech GmbH (Marktredwitz, Germany), LGC Promochem GmbH (Wesel, Germany), RomerLabs^®^ (Tulln Austria), Sigma-Aldrich (Vienna, Austria), and Toronto Research Chemicals (Toronto, ON, Canada). Stock solutions were purchased or prepared by dissolving a solid standard in water, acetonitrile, methanol, or their mixtures. All stock solutions were stored at −20 °C and allowed to reach room temperature prior to usage.

### 4.2. Sample Extraction

All samples were weighed in a 50 mL Falcon tube (5.00 g each) and extracted with 40 mL of extraction solvent (acetonitrile/water/acetic acid 79:20:1, *V*/*V*/*V*). After the addition of the extraction solvent, the tubes were vortexed to allow separation of the hop pellets and ensure proper extraction. The extraction was carried out on a GFL 3017 rotary shaker (GFL; Burgwedel, Germany) for 90 min, at 180 rpm at room temperature. After extraction, the tubes were allowed to settle and 1 mL of the extract was transferred to an Eppendorf tube where the fatty layer was separated by pipetting 500 µL of the bottom layer to an autosampler vial and diluted with 500 µL of dilution solvent (acetonitrile/water/acetic acid 20:79:1, *V*/*V*/*V*). The samples were analyzed without any further manipulation according to the state-of-the-art LC-MS/MS multi-toxin method described by Sulyok et al. (2020) [62]. The samples were analyzed on an Agilent 1290 UHPLC system (Agilent Technologies, Waldbronn, Germany), coupled to a Sciex QTrap 5500 MS/MS equipped with an ESI source (TurboV) (Sciex, Foster City, CA, USA). The chromatographic separation was performed on a Gemini C18 column (150 × 4.6 mm i.d., 5 μm filling particle size), and a C18 security guard cartridge (4 × 3 mm i.d., 5 μm filling particle size) (Phenomenex, Torrance, CA, USA). The used eluents were as follows: eluent A: methanol:water:acetic acid (10:89:1, *V*/*V*/*V*); eluent B: methanol:water:acetic acid (97:2:1, *V*/*V*/*V*). All of the further method details are described in the method paper published by Sulyok et al., (2020) [62].

### 4.3. Statistical Analysis

For the statistical analysis, Statistica 14.1.0.8. (Cloud Software Group Inc.) and Tableau Desktop 2023.3.1 (Tableau Software) were used. For the normality of the data distribution the Shapiro–Wilk W test was used, and for further comparison of the data distribution non-parametric tests were used (Mann–Whitney U test, and Kruskal–Wallis ANOVA), while for the correlations the Spearman rank-order test was used. Tableau was used to generate heat maps based on the average concentrations in the samples from different Croatian counties. All data below LOD values were substituted with 0 in accordance with the lower bound EFSA guide on the management of left-censored data [63].

## Data Availability

Dataset available on request from the authors.
